# Bidirectional association between thyroid dysfunction and depressive symptoms: the role of levothyroxine therapy and shared molecular mechanisms

**DOI:** 10.1186/s12991-026-00694-x

**Published:** 2026-08-16

**Authors:** Han-xin Fu, Jia-xin Wang, Bin Qian, Xin Zhang, Lin-lin Hu

**Affiliations:** 1 Department of Clinical Psychology, Hangzhou TCM Hospital Affiliated to Zhejiang Chinese Medical University, Hangzhou, 310007 Zhejiang China; 2 School of Pharmaceutical Sciences, Zhejiang Chinese Medical University, Hangzhou, 310053 Zhejiang China

**Keywords:** Thyroid dysfunction, Hypothyroidism, Depressive symptoms, Depression, Levothyroxine

## Abstract

**Background:**

The association between thyroid dysfunction, thyroid hormone replacement therapy, and depression remains inconsistent. This study investigated the bidirectional risk association between thyroid dysfunction and depressive symptoms, focusing on the effect of thyroid hormone therapy on depressive symptoms, and exploring the shared molecular basis between hypothyroidism treated with levothyroxine and depression at the genetic level.

**Methods:**

Part one utilized 2007–2012 National Health and Nutrition Examination Surveys (NHANES) data to analyze associations between thyroid profiles and depressive symptoms. Part two compared molecular expression profiles between levothyroxine-treated hypothyroid patients and major depressive disorder patients using GEO datasets (GSE251778, GSE103305).

**Results:**

Part one revealed a bidirectional association between depressive symptoms and hypothyroidism: patients with decreased interest, appetite changes, or feelings of worthlessness/failure were more likely to have hypothyroidism, while hypothyroidism was associated with an increased risk of depressive symptoms in general (PHQ-9 score ≥ 5). Compared with those with normal thyroid function, participants receiving thyroid hormone replacement therapy had a significantly higher risk of PHQ-9 score ≥ 5 (OR = 1.328; 95% CI: 1.115–1.582; *P* = 0.002), whereas untreated patients with elevated TSH did not (OR = 0.893; 95% CI: 0.589–1.356; *P* = 0.597). After adjusting for confounders, the difference in depressive symptoms risk between the treated and untreated groups was of marginal significance (OR = 1.486, 95% CI: 0.956–2.311; *P* = 0.078). Part two identified 17 common differentially expressed genes (DEGs) between levothyroxine-treated hypothyroid patients and major depressive disorder patients by intersecting their respective DEGs with controls. GO enrichment analysis revealed significant terms such as ‘oxidoreductase activity, acting on NAD(P)H, quinone or similar compound as acceptor’ and ‘oxidoreductase activity, acting on NAD(P)H’. KEGG enrichment analysis revealed pathways related to antigen processing and presentation, chemical carcinogenesis-reactive oxygen species, and the cytoskeleton in muscle cells. PPI network analysis revealed that IFNG is the core gene.

**Conclusions:**

This study confirmed the bidirectional association between thyroid disorders and depressive symptoms. The results also suggest that levothyroxine replacement therapy may be associated with an increased risk of depressive symptoms in hypothyroid patients. Potential molecular mechanisms involved an imbalance in redox homeostasis and abnormal interferon-γ signalling pathways. These findings provide new insights into the pathophysiological mechanisms linking thyroid dysfunction and depression and may offer clues for future therapeutic strategies.

## Introduction

Thyroid hormones play key roles in brain development and neurocognitive function [1, 2]. Thyroid dysfunction can induce a variety of neuropsychiatric manifestations and is associated with the development of neuropsychiatric disturbances such as anxiety, schizophrenia, and depression [3, 4]. Currently, the link between depression and thyroid dysfunction is beginning to attract increased attention.

Depression is a mood disorder characterized by a persistent feeling of sadness and/or an inability to experience pleasure, with associated deficits in daily functioning [5]. It is among the most common psychiatric conditions and is a leading cause of disability and loss of productivity worldwide [5, 6]. Studies have reported a positive genetic correlation between hypothyroidism and major depressive disorder (MDD), with MDD having a causal effect on increasing the risk of hypothyroidism [7]. Conversely, hypothyroidism is also considered a potential important factor in the pathogenesis of depression [8]. For example, a cross-sectional study revealed that approximately 59% of patients with hypothyroidism present with depressive symptoms [9].

Based on this bidirectional relationship, thyroid hormones, particularly triiodothyronine (LT3), are sometimes utilized as an adjunctive treatment for depression in clinical practice, serving the purpose of accelerating onset and enhancing efficacy [10, 11]. Furthermore, studies have also shown that add-on treatment with supraphysiologic doses of levothyroxine (LT4) has shown efficacy in acute refractory unipolar depression and represents a promising strategy for depressive mood disorders [11].

However, some studies have reported the opposite results. Some studies report no significant association, whereas others report conflicting results, including both positive and negative correlations [7, 12–15]. One literature review on the use of thyroid hormones as an adjunctive therapy for treatment-resistant depression reported that the efficacy of these agents was not significantly superior to that of placebos or lithium [16]. In our clinical practice, we have also observed that patients with hypothyroidism continue to experience significant emotional problems and sleep disturbances despite receiving levothyroxine (LT4) treatment. In addition, the improvement in these symptoms tended to be less favourable in hypothyroid patients who received medication than in those who did not receive medication. However, this view currently lacks supporting clinical research evidence.

The association between thyroid dysfunction and depression has yielded complex and inconsistent findings in existing studies. Therefore, this study was aimed at elucidating, through a large-sample analysis, the complex relationship between thyroid dysfunction and depressive symptoms. Given that LT4 is the preferred treatment for hypothyroidism, we will specifically focus on patients with hypothyroidism currently receiving LT4 treatment.

In the current study, we conducted a cross-sectional analysis using data from the 2007–2012 National Health and Nutrition Examination Surveys (NHANES) to investigate the associations between PHQ-9-based depressive symptoms and thyroid dysfunction in the U.S. adult population. We subsequently analysed the effect of thyroid hormone replacement therapy on the risk of depressive symptoms in patients with hypothyroidism. In addition, we identified common differentially expressed genes (DEGs) through the Gene Expression Omnibus (GEO) database by comparing patients with depression and patients with hypothyroidism who were receiving LT4 replacement therapy. Through Gene Ontology (GO) and Kyoto Encyclopedia of Genes and Genomes (KEGG) pathway enrichment analyses, as well as protein-protein interaction (PPI) network analysis, we revealed the potential biological processes and mechanisms of both. Through this comprehensive study, we hope to elucidate the interplay between thyroid dysfunction and depressive symptoms more clearly and provide new insights to use for related treatments.

## Methods

This study integrated data from two sources: the NHANES and the GEO database.

### NHANES data analysis

#### Data collection

The NHANES is a nationally representative health survey in the United States that includes health exams and laboratory tests for participants of all ages. The 2007–2012 NHANES is the only available six-year module that includes extensive demographic data, questionnaire surveys, laboratory tests and prescription medication lists. Our study included all participants aged 20 years or older who completed the PHQ-9 questionnaire, reported thyroid medication usage, and had available thyroid-stimulating hormone (TSH) test results.

#### Variable definitions

To account for potential confounding, we included the following covariates: age, sex, race/ethnicity, education level, body mass index (BMI), marital status, smoking status, family income-to-poverty ratio, exercise, and history of liver disease, cardiovascular disease, chronic kidney disease (CKD), and diabetes. Participant race/ethnicity was categorized into four groups: non-Hispanic black, non-Hispanic white, Mexican-American, or other. Education level was categorized into five groups: less than 9th grade, 9th-11th grade with no diploma, high school graduate or equivalent, some college, and college graduate or higher. BMI was categorized into four groups: underweight (< 18.5 kg/m²), normal (18.5 to < 25 kg/m²), overweight (25 to < 30 kg/m²), and obese (≥ 30 kg/m²). Marital status was categorized into three groups: married or living with a partner; widowed, divorced, or separated; or never married. Smoking status was categorized into three groups: never smokers (< 100 lifetime cigarettes), former smokers (> 100 lifetime cigarettes but not current smokers), and current smokers. Exercise was categorized into three groups: vigorous activity, defined as activities causing large increases in breathing or heart rate for at least 10 min continuously; moderate activity, defined as activities causing small increases in breathing or heart rate for at least 10 min continuously; or sedentary, defined as mostly sitting or reclining. Hypertension was defined as a previous diagnosis, or as systolic blood pressure ≥ 140 mmHg or diastolic blood pressure ≥ 90 mmHg. Diabetes and liver disease were each defined as a previous diagnosis. CKD was defined as an estimated glomerular filtration rate (eGFR) < 60 mL/min/1.73 m². The eGFR was calculated using the CKD Epidemiology Collaboration (CKD-EPI) equation [17].

#### Diagnosis of thyroid condition based on laboratory results and medication data

**Fig. 1:**
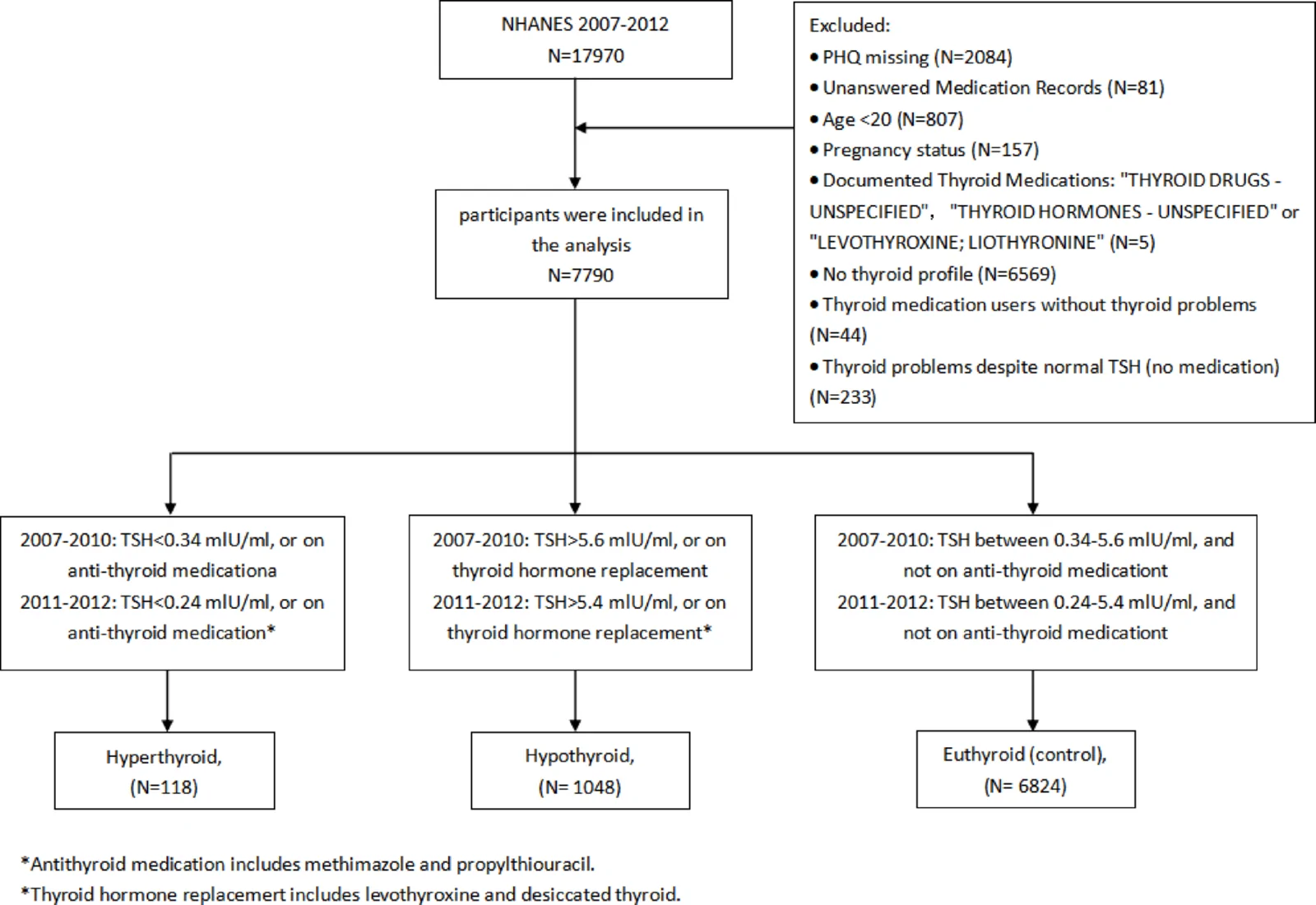
Participant Flowchart for Thyroid Dysfunction Subgroups in Study 1 (NHANES)

This study referenced the manufacturer’s guidelines provided in the NHANES documentation to determine the normal reference ranges for TSH based on different survey cycles. For the 2007–2008 and 2009–2010 cycles, the TSH reference range was 0.34 to 5.6 mIU/mL; for the 2011–2012 cycle, it ranged from 0.24 to 5.4 mIU/mL [18–20]. In accordance with previous literature, thyroid function was determined based on self-reported use of thyroid-related medications and TSH test results [21]. Hypothyroidism was defined as follows: (1) participants who reported having been informed of a thyroid problem and were using LT4 or dried thyroid (DT), regardless of their TSH level, or (2) participants whose TSH level was greater than the upper limit of the normal range and who were not taking thyroid hormone replacement medication. Hyperthyroidism was defined as (1) participants who reported having been informed of a thyroid problem and were using methimazole or propylthiouracil, regardless of their TSH level, or (2) participants whose TSH level was under the lower limit of the normal range and who were not taking antithyroid medication. Euthyroidism (normal thyroid function) was defined as having no thyroid problems, not taking thyroid hormone replacement or antithyroid medication, and having a TSH level within the normal range (Fig. 1).

Normal FT4 reference ranges were derived from the manufacturer’s guidelines provided in the NHANES documentation, which ranged from 0.6 to 1.6 ng/dL [22–24]. To characterize the patients with hypothyroidism and hyperthyroidism, we referenced the relevant literature and then categorized them on the basis of their FT4 values [21]. We examined the prevalence of the following conditions and analysed their association with depressive symptoms: well-controlled hypothyroidism (normal TSH, normal FT4, and currently on medication), well-controlled hyperthyroidism (normal TSH, normal FT4, and currently on medication), poorly controlled hypothyroidism (abnormal TSH or abnormal FT4, and currently on medication), poorly controlled hyperthyroidism(abnormal TSH or abnormal FT4, and currently on medication), overt hypothyroidism(high TSH, low FT4, and without medication), overt hyperthyroidism (low TSH, high FT4, and without medication), subclinical hypothyroidism (high TSH, normal FT4, and without medication), and subclinical hyperthyroidism (low TSH, normal FT4, and without medication). Participants with hypothyroidism or hyperthyroidism who lacked both FT4 and TSH test results were classified into the “Other” category.

#### Measurement of depressive symptoms

**Fig. 2:**
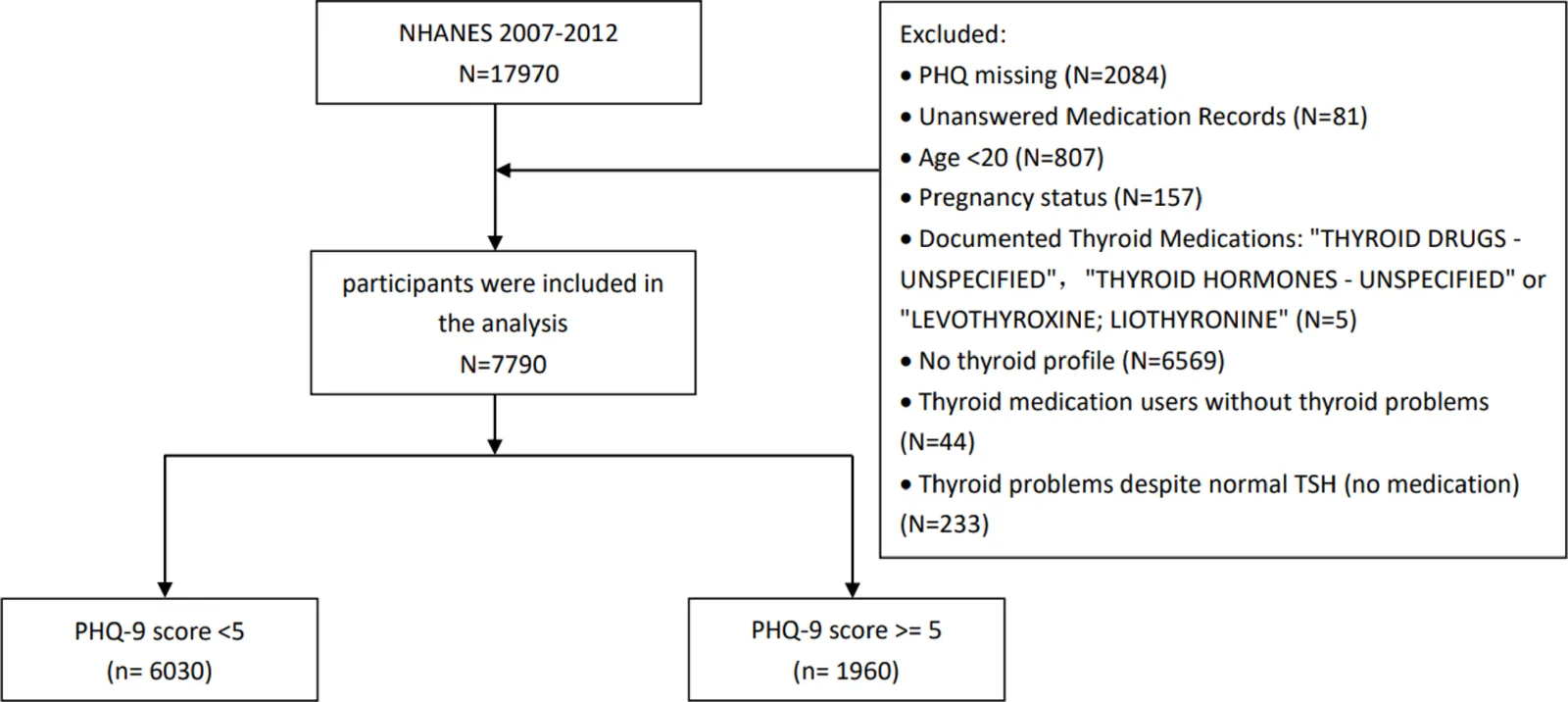
Participant Flowchart for Depressive Symptoms Subgroups in Study 1 (NHANES)

The PHQ-9 was used to assess depressive symptoms experienced over the past two weeks. This questionnaire comprises nine items, each evaluated on a four-point scale: 0 (not at all), 1 (several days), 2 (more than half the days), and 3 (nearly every day). Five of these items address cognitive and emotional symptoms, such as “Have little interest in doing things” and “Thought you would be better off dead.” The remaining four items evaluate somatic symptoms, including “Trouble sleeping or sleeping too much” and “Poor appetite or overeating.” Based on the PHQ-9, a score of ≥ 5 was defined as a positive screen for depressive symptoms, with severity categorized as mild (≥ 5), moderate (≥ 10), and moderately severe/severe (≥ 15) (Fig. 2) [25]. For the subsequent analysis of specific depressive symptoms, we dichotomized the scores of each item: scores of 0 or 1 were coded as the absence of symptoms, and scores of 2 or 3 were coded as the presence of symptoms. Specifically, for the “Thought you would be better off dead” item, responses of 1, 2, or 3 were all coded as indicating the presence of the symptom.

#### Statistical analysis

Statistical analyses of the NHANES data were performed using R within the RStudio integrated development environment. In the description of population characteristics, continuous variables were analysed using t tests, and categorical variables were analysed using chi-square tests. Binary logistic regression models were used to assess the independent effects of depressive symptoms and their specific manifestations on the risk of hypothyroidism/hyperthyroidism, as well as the independent effects of the aforementioned thyroid disorders on the risk of developing depressive symptoms, aiming to elucidate the bidirectional association between them. The proposed models included Model 1 (unadjusted), Model 2 (adjusted for a subset of covariates), and Model 3 (adjusted for all covariates). Statistical significance was set at *P* < 0.05.

### GEO data analysis

#### Identification of gene expression datasets

The GEO is a public repository for storing gene expression data, microarray data, and other high-throughput functional genomics data. The GSE251778 and GSE103305 datasets were downloaded from the GEO database using RStudio. Most dataset processing and analysis were conducted in RStudio. The GSE251778 dataset (depression-associated transcriptomes, GPL20301 and GPL24676 platforms) contains whole blood samples from 80 patients diagnosed with MDD and 89 healthy controls. MDD was defined as having a HAMD score > = 19 [26]. The GSE103305 dataset (hypothyroidism-related transcriptomes, GPL16791 platform) consists of whole blood samples collected from 8 hypothyroidism patients before and after LT4 treatment. Based on the sample origin, we divided the participants into three groups: the LT4-treated hypothyroidism group, the MDD group, and the healthy control group.

#### Differentially expressed genes and co-expressed genes

First, we read the downloaded high-throughput count data into RStudio. Next, the count data were annotated, and gene symbols were converted using methods such as retrieving gene annotation information from the Ensembl database via the biomaRt package. Rows with missing values or lacking gene symbols were removed. The expression data for any remaining duplicate rows (identified by the gene symbol) were then summed. The threshold for filtering out genes with low expression was set to at least 5 reads across 60% of the samples. Differential gene expression analysis was performed using DESeq2, yielding DEGs between the LT4-treated hypothyroidism group and the healthy control group, as well as between the MDD group and the healthy control group. DEGs were defined as those with an adjusted P-value < 0.05 and |log2(fold change)| > 0.5. Lastly, based on the log2(fold change) values, we extracted upregulated and downregulated genes from each of the two differential gene expression datasets. We subsequently identified their common DEGs through intersection analysis. These intersecting results were visualized using Venn diagrams.

#### Functional enrichment analysis

Using the “clusterProfiler” R package, we performed GO and KEGG pathway enrichment analyses on the common DEGs that were shared between the DEG sets from the ‘LT4-treated hypothyroidism vs. healthy control’ comparison and the ‘major depressive disorder vs. healthy control’ comparison. These analyses revealed signalling pathways that may underlie shared biological mechanisms between hypothyroidism (after LT4 treatment) and depression. The enrichment results were then visualized using the ggplot2 R package.

#### PPI network analysis

PPI networks are used to analyse the interactions of proteins within biological systems systematically. This approach is valuable for elucidating the mechanisms underlying biological signalling and energy metabolism under specific physiological conditions, such as disease, and for understanding the functional relationships between proteins. Gene modules identified through GO and KEGG enrichment analyses were imported into the Search Tool for the Retrieval of Interacting Genes/Proteins (STRING) database for PPI network construction and visualization, and *Homo sapiens* was used as the species for analysis.

## Results

### Population characteristics/Baseline characteristics of participants

The total number of participants in the 2007 to 2012 NHANES was 17,970. Only 7790 participants met the inclusion criteria and were analysed: 118 were identified as hyperthyroid, 6824 were euthyroid, and 1048 were hypothyroid. The demographic and health characteristics of the hypothyroid and control (euthyroid) participants are listed in Table 1. Compared with the euthyroid group, individuals with hypothyroidism were more likely to be female, older, non-Hispanic White, more highly educated, widowed/divorced/separated, obese, and former smokers. The demographic and health characteristics of the hyperthyroid and control (euthyroid) participants are listed in Table 2. Compared with individuals in the euthyroid group, individuals in the hyperthyroidism group were more likely to be female, non-Hispanic Black, of lighter weight, and current smokers.

**Table 1:** Sample Characteristics of Hypothyroid and Control (Euthyroid) Participants

	euthyroid	hypothyroid	*p*-value
	(*n* = 6824)	(*n* = 1048)	
Sex			< 0.001
Male	3685(54.0)	257(24.5)	
Female	3139(46.0)	791(75.5)	
Age, mean(SD)	48.9(17.5)	61.7(14.9)	< 0.001
Ethnicity			< 0.001
Hispanic	1897(27.8)	206(19.7)	
Non-hispanic white	3073(45.0)	697(66.5)	
Non-hispanic black	1418(20.8)	100(9.5)	
Other	436(6.4)	45(4.3)	
Education			0.001
Less than 9th	798(11.7)	102(9.8)	
9-11th grade	1142(16.7)	136(13.0)	
High school or	1605(23.5)	244(23.3)	
equivalent			
Some college	1874(27.5)	309(29.6)	
College or higher	1401(20.5)	254(24.3)	
BMI status			< 0.001
Underweight	103(1.5)	12(1.2)	
Normal	1884(27.9)	241(23.2)	
Overweight	2322(34.4)	331(31.9)	
Obese	2447(36.2)	453(43.7)	
Smoking status			< 0.001
Never	3604(52.8)	589(56.3)	
Former	1691(24.8)	328(31.3)	
Current	1526(22.4)	130(12.4)	
Marital			< 0.001
Married, or living	4082(59.8)	625(59.7)	
with partner			
Widowed, divorced,	1483(21.7)	350(33.4)	
or separated			
Never married	1258(18.4)	72(6.9)	
PHQ-9,mean(SD)	3.2(4.3)	3.6(4.4)	0.003
Depression			0.001
PHQ-9 < 5	5203(76.2)	750(71.6)	
PHQ-9 ≥ 5	1621(23.8)	298(28.4)	
Depression classification			0.006
No depression	5203(76.2)	750(71.6)	
Mild depression	1011(14.8)	184(17.6)	
Moderate depression	387(5.7)	79(7.5)	
Moderately severe/severe	223(3.3)	35(3.3)	
depression			
Depressive symptoms			
Have little interest	502(7.4)	89(8.5)	0.194
Feeling down	515(7.5)	88(8.4)	0.335
Trouble sleeping	1102(16.1)	208(19.8)	0.003
Feeling tired	1093(16.0)	208(19.8)	0.002
Appetite change	589(8.6)	123(11.7)	0.001
Feeling bad about	388(5.7)	72(6.9)	0.128
self			
Trouble concentrating	382(5.6)	62(5.9)	0.678
Moving/speaking	253(3.7)	38(3.6)	0.896
slowly			
Better off dead	281(4.1)	33(3.1)	0.136

**Table 2:** Sample Characteristics of Hyperthyroid and Control (Euthyroid) Participants

	euthyroid	hyperthyroid	*p*-value
	(*n* = 6824)	(*n* = 118)	
Sex			0.001
Male	3685(54.0)	46(39.0)	
Female	3139(46.0)	72(61.0)	
Age, mean(SD)	48.9(17.5)	48.9(18.2)	0.998
Ethnicity			
Hispanic	1897(27.8)	33(28.0)	0.032
Non-hispanic white	3073(45.0)	42(35.6)	
Non-hispanic black	1418(20.8)	37(31.4)	
Other	436(6.4)	6(5.1)	
Education			0.377
Less than 9th	798(11.7)	17(14.4)	
9-11th grade	1142(16.7)	24(20.3)	
High school or	1605(23.5)	25(21.2)	
equivalent			
Some college	1874(27.5)	35(29.7)	
College or higher	1401(20.5)	17(14.4)	
BMI status			0.030
Underweight	103(1.5)	5(4.2)	
Normal	1884(27.9)	40(33.9)	
Overweight	2322(34.4)	36(30.5)	
Obese	2447(36.2)	35(29.7)	
Smoking status			< 0.001
Never	3604(52.8)	48(40.7)	
Former	1691(24.8)	25(21.2)	
Current	1526(22.4)	45(38.1)	
Marital			0.994
Married, or living	4082(59.8)	70(59.3)	
with partner			
Widowed, divorced,	1483(21.7)	26(22.0)	
or separated			
Never married	1258(18.4)	22(22.0)	
PHQ-9,mean(SD)	3.2(4.3)	3.9(4.5)	0.091
Depression			0.008
PHQ-9 < 5	5203(76.2)	77(65.3)	
PHQ-9 ≥ 5	1621(23.8)	41(34.7)	
Depression classification			0.035
No depression	5203(76.2)	77(65.3)	
Mild depression	1011(14.8)	28(23.7)	
Moderate depression	387(5.7)	8(6.8)	
Moderately severe/severe	223(3.3)	5(4.2)	
depression			
Depressive symptoms			
Have little interest	502(7.4)	9(7.6)	0.911
Feeling down	515(7.5)	12(10.2)	0.286
Trouble sleeping	1102(16.1)	22(18.6)	0.466
Feeling tired	1093(16.0)	27(22.9)	0.044
Appetite change	589(8.6)	13(11.0)	0.361
Feeling bad about	388(5.7)	6(5.1)	0.780
self			
Trouble concentrating	382(5.6)	6(5.1)	0.810
Moving/speaking	253(3.7)	7(5.9)	0.214
slowly			
Better off dead	281(4.1)	7(5.9)	0.344

Table 3 presents the characteristics of the hypothyroid and hyperthyroid populations, which are based on thyroid medication use, TSH levels, and FT4 levels. Owing to the nationally representative nature of the NHANES, we could estimate the actual size of each group within the larger U.S. adult population from 2007 to 2012. Those identified as hypothyroid accounted for 13% of the population, while those identified as hyperthyroid accounted for 1.5%. Approximately 86% of the hypothyroid patients were taking LT4 (approximately 98%) or DT, and approximately 14% of the hyperthyroid patients were taking methimazole or propylthiouracil. Fewer than half of the patients receiving thyroid hormone replacement therapy had normal thyroid function at the time of blood draw. However, this conclusion may be inaccurate because of the substantial proportion of patients with missing TSH data. Among untreated participants with hypothyroidism, approximately 18% exhibited overt hypothyroidism, while 82% presented with subclinical hypothyroidism. Participants identified as having hyperthyroidism made up 1.5% of the overall population, approximately one-ninth the proportion of the U.S. population with hypothyroidism. Approximately 19% of patients treated with anti-thyroid medications exhibited normal thyroid function at the time of blood draw. Among untreated participants with hyperthyroidism, approximately 13% were classified as having overt hyperthyroidism, while 87% were classified as having subclinical hyperthyroidism. This distribution may reflect under-recognition of subclinical hypothyroidism or hyperthyroidism or be influenced by clinical management decisions.

**Table 3:** Characteristics of Hypothyroid and Hyperthyroid Populations, Based on Thyroid Medications, TSH, and FT4

	*N*	Weighted
		Percentage
Total hypothyroidism	1048	13.12
Taking hypothyroid medication	902	11.30
Not taking hypothyroid medication	146	1.83
Total hyperthyroidism	118	1.48
Taking hyperthyroid medication	16	0.20
Not taking hyperthyroid medication	102	1.28
Total euthyroid (control group)	6824	85.41
Total	7990	100.00
Among hypothyroid population	1048	
On medication		
Controlled (normal TSH)	370	35.31
Uncontrolled (abnormal TSH)	128	12.21
Other (unknown TSH)	404	38.55
Not on medication		
Overt (high TSH, low FT4)	26	2.48
Subclinical (high TSH, normal FT4)	120	11.45
Among hyperthyroid population	118	
On medication		
Controlled (normal TSH)	3	2.54
Uncontrolled (abnormal TSH)	4	3.39
Other (unknown TSH)	9	7.63
Not on medication		
Overt (low TSH, high FT4)	13	11.02
Subclinical (low TSH, normal FT4)	89	75.42

### Relationships between hypothyroidism/hypothyroid and depressive symptoms

Table 4 presents the logistic regression analysis of hypothyroidism and depressive symptoms. Hypothyroidism was positively associated with the PHQ-9 sum score (OR = 1.029; 95% CI: 1.012–1.046; *P* = 0.001) after adjustment for age, sex, race/ethnicity, education, BMI, smoking status and marital status. Compared with control patients, patients with a PHQ-9 score ≥ 5 had a significantly increased risk of developing hypothyroidism (OR = 1.345; 95% CI: 1.140–1.587; *P* < 0.001). Specifically, compared with patients without depressive symptoms, patients with mild depression were 1.263 times more likely to have hypothyroidism, and patients with moderate depression were 1.679 times more likely to have hypothyroidism. Furthermore, hypothyroidism was associated with specific symptoms of depression, both when examined separately and when adjusted for all other depressive symptoms. Specifically, compared to the control group, patients with decreased interest, appetite changes (i.e., poor appetite or overeating), or feelings of worthlessness/failure were 1.309, 1.483, and 1.369 times more likely, respectively, to develop hypothyroidism.

**Table 4:** Logistic regression analysis of Hypothyroid and Depressive Symptoms

	Model 1^a^		Model 2^b^		Model 3^c^	
	OR (CI)	*p*-value	OR (CI)	*p*-value	OR (CI)	*p*-value
PHQ-9 total scores	1.022(1.008–1.037)	0.003	1.028(1.012–1.045)	< 0.001	1.029(1.012–1.046)	0.001
Depression						
PHQ-9 < 5	1.000		1.000		1.000	
PHQ-9 ≥ 5	1.275(1.103–1.475)	0.001	1.357(1.156–1.593)	< 0.001	1.345(1.140–1.587)	< 0.001
Depression severity						
No depression	1.000		1.000		1.000	
Mild depression	1.263(1.060–1.504)	0.009	1.290(1.066–1.562)	0.009	1.263(1.039–1.534)	0.019
Moderate depression	1.416(1.099–1.825)	0.007	1.676(1.272–2.209)	< 0.001	1.679(1.262–2.235)	< 0.001
Moderately severe/severe	1.089(0.756–1.568)	0.647	1.163(0.790–1.710)	0.444	1.229(0.829–1.821)	0.304
depression						
Depressive symptoms						
Have little interest	1.169(0.924–1.479)	0.194	1.280(0.993–1.650)	0.057	1.309(1.011–1.695)	0.041
Feeling down	1.123(0.887–1.422)	0.336	1.174(0.910–1.514)	0.217	1.204(0.929–1.560)	0.160
Trouble sleeping	1.286(1.090–1.516)	0.003	1.217(1.017–1.457)	0.032	1.174(0.976–1.413)	0.089
Feeling tired	1.298(1.101–1.531)	0.002	1.194(0.998–1.427)	0.052	1.192(0.993–1.431)	0.060
Appetite change	1.408(1.145–1.730)	0.001	1.491(1.190–1.869)	0.001	1.483(1.175–1.870)	0.001
Feeling bad about self	1.224(0.943–1.588)	0.129	1.356(1.023–1.797)	0.034	1.369(1.025–1.828)	0.033
Trouble concentrating	1.060(0.804–1.398)	0.678	1.190(0.886-1.600)	0.248	1.216(0.899–1.644)	0.204
Moving/speaking slowly	0.977(0.690–1.383)	0.896	1.185(0.818–1.718)	0.370	1.211(0.831–1.766)	0.319
Better off dead	0.757(0.525–1.092)	0.137	0.826(0.558–1.221)	0.337	0.873(0.585–1.304)	0.508

a Model 1: no covariates were adjusted

b Model 2: age, sex, race/ethnicity, education were adjusted

c Model 3: BMI, smoke, marital and variables from model 2 were adjusted

Table 5 presents the logistic regression analysis of hyperthyroidism and depressive symptoms. Compared with patients without depressive symptoms, patients with mild depression were 1.716 times more likely to have hyperthyroidism after adjustments for age, sex, race/ethnicity, education, BMI, smoking status and marital status. Hyperthyroidism was not associated with any of the specific symptoms of depression when examined separately or when adjusted for all the other symptoms of depression.

**Table 5:** Logistic regression analysis of Hyperthyroid and Depressive Symptoms

	Model 1^a^		Model 2^b^		Model 3^c^	
	OR (CI)	*p*-value	OR (CI)	*p*-value	OR (CI)	*p*-value
PHQ-9 total scores	1.034(0.996–1.073)	0.077	1.020(0.982–1.060)	0.302	1.007(0.966–1.049)	0.748
Depression						
PHQ-9 < 5	1.000		1.000		1.000	
PHQ-9 ≥ 5	1.709(1.165–2.507)	0.006	1.526(1.030–2.261)	0.035	1.437(0.956–2.160)	0.082
Depression severity						
No depression	1.000		1.000		1.000	
Mild depression	1.871(1.208–2.899)	0.005	1.727(1.108–2.691)	0.016	1.716(1.093–2.695)	0.019
Moderate depression	1.397(0.670–2.914)	0.373	1.206(0.573–2.539)	0.622	1.093(0.513–2.329)	0.818
Moderately severe/severe	1.515(0.607–3.781)	0.373	1.216(0.482–3.069)	0.679	0.881(0.312–2.485)	0.810
depression						
Depressive symptoms						
Have little interest	1.040(0.524–2.064)	0.911	0.900(0.450–1.797)	0.764	0.851(0.424–1.710)	0.651
Feeling down	1.387(0.758–2.536)	0.288	1.190(0.645–2.198)	0.577	1.015(0.532–1.936)	0.964
Trouble sleeping	1.190(0.745-1.900)	0.466	1.066(0.664–1.712)	0.792	0.963(0.590–1.573)	0.881
Feeling tired	1.556(1.008–2.402)	0.046	1.383(0.890–2.150)	0.150	1.271(0.806–2.003)	0.303
Appetite change	1.311(0.732–2.346)	0.363	1.098(0.608–1.980)	0.757	0.935(0.503–1.739)	0.832
Feeling bad about self	0.889(0.388–2.033)	0.780	0.789(0.343–1.816)	0.578	0.592(0.237–1.481)	0.263
Trouble concentrating	0.903(0.395–2.067)	0.810	0.791(0.344–1.819)	0.581	0.746(0.322–1.729)	0.494
Moving/speaking slowly	1.638(0.755–3.552)	0.212	1.455(0.665–3.182)	0.348	1.070(0.458–2.504)	0.875
Better off dead	1.468(0.678–3.181)	0.330	1.349(0.618–2.944)	0.452	1.209(0.548–2.665)	0.638

a Model 1: no covariates were adjusted

b Model 2: age, sex, race/ethnicity, education were adjusted

c Model 3: BMI, smoke, marital and variables from model 2 were adjusted

### Relationship between depressive symptoms and thyroid disease

When stratified by the total PHQ-9 score, the 7790 adult participants included 6030 with a score of < 5 and 1960 with a score of ≥ 5. Table 6 presented the demographic and health characteristics of these two groups, which differed significantly across most covariates. The prevalence of depressive symptoms was greater among females, younger individuals, Hispanic individuals, those with lower levels of education, those who were widowed/divorced/separated or never married, those who were obese, those who were current smokers, those with a lower family income-to-poverty ratio, those who exercised less, and those with liver disease, cardiovascular disease, or diabetes than among their respective counterparts.

**Table 6:** Sample Characteristics of the study population based on PHQ-9 score

	PHQ-9 score	PHQ-9 score	*p*-value
	(< 5)	(≥ 5)	
Overall	6030	1960	
Sex			< 0.001
Male	3220(53.4)	768(39.2)	
Female	2810(46.6)	1192(60.8)	
Age, mean(SD)	51.06(17.90)	49.01(17.00)	< 0.001
Ethnicity			0.003
Hispanic	1562(25.9)	574(29.3)	
Non-hispanic white	2880(47.8)	932(47.6)	
Non-hispanic black	1198(19.9)	357(18.2)	
Other	390(6.5)	97(4.9)	
Education			< 0.001
Less than 9th	633(10.5)	284(14.5)	
9-11th grade	892(14.8)	410(20.9)	
High school or equivalent	1424(23.6)	450(23.0)	
Some college	1655(27.4)	563(28.7)	
College or higher	1421(23.6)	251(12.8)	
BMI status			< 0.001
Underweight	83(1.4)	37(1.9)	
Normal	1691(28.0)	474(24.2)	
Overweight	2155(35.7)	534(27.2)	
Obese	2048(34.0)	887(45.3)	
Smoking status			< 0.001
Never	3344(55.5)	897(45.8)	
Former	1588(26.3)	456(23.3)	
Current	1096(18.2)	605(30.9)	
Marital			< 0.001
Married, or living with partner	3788(62.8)	989(50.5)	
Widowed, divorced, or separated	1262(20.9)	597(30.5)	
Never married	979(16.2)	373(19.0)	
Poverty income ratio	2.72(1.62)	2.02(1.51)	< 0.001
Exercise			< 0.001
Sedentary	3037(50.36)	1298(66.22)	
Moderate activity	1665(27.61)	403(20.56)	
Vigorous activity	1328(22.02)	259(13.21)	
Liver disease			< 0.001
Yes	175(2.90)	113(5.77)	
No	5848(96.98)	1843(94.03)	
Cardiovascular disease			< 0.001
Yes	2619(43.43)	988(50.41)	
No	3348(55.52)	953(48.62)	
Chronic kidney disease			0.336
Yes	485(8.04)	144(7.35)	
No	5513(91.43)	1800(91.84)	
Diabetes			< 0.001
Yes	680(11.28)	327(16.68)	
No	5345(88.64)	1633(83.32)	
Thyroid status			< 0.001
Euthyroid	5203(86.3)	1621(82.7)	
Hyperthyroid	77(1.3)	41(2.1)	
Hypothyroid with taking medication	636(10.5)	266(13.6)	
Hypothyroid without taking	114(1.9)	32(1.6)	
medication			

The data in Table 7 demonstrate that thyroid function differed between participants with PHQ-9 scores < 5 and those with scores ≥ 5. After adjustments for all covariates listed in the table, compared with normal thyroid function, hypothyroidism was significantly associated with an increased risk of a PHQ-9 score ≥ 5 (OR = 1.256; 95% CI: 1.065–1.480; *P* = 0.007).

**Table 7:** Logistic Regression ORs of a Self-Reported Diagnosis of Depressive Symptoms

	Model 1^a^		Model 2^b^		Model 3^c^	
	OR(CI)	*p*-value	OR(CI)	*p*-value	OR (CI)	*p*-value
Thyroid status						
Euthyroid	1.000		1.000		1.000	
Hyperthyroid	1.709(1.165–2.507)	0.006	1.470(0.985–2.194)	0.060	1.440(0.959–2.161)	0.078
Hypothyroid	1.275(1.103–1.475)	0.001	1.265(1.077–1.486)	0.004	1.256(1.065–1.480)	0.007
Based on medication status						
Euthyroid	1.000		1.000		1.000	
Hyperthyroid	1.709(1.165–2.507)	0.006	1.471(0.985–2.196)	0.059	1.441(0.960–2.162)	0.078
Hypothyroid with taking	1.342(1.151–1.565)	< 0.001	1.338(1.127–1.588)	0.001	1.328(1.115–1.582)	0.002
medication						
Hypothyroid without	0.901(0.606–1.339)	0.606	0.896(0.593–1.353)	0.602	0.893(0.589–1.356)	0.597
taking medication						

a Model 1: No covariates were adjusted

b Model 2: Adjusted for age, sex, race/ethnicity, education, BMI, smoke, and marital

c Model 3: Adjusted for variables in Model 2 plus family income-to-poverty ratio, exercise, liver disease, cardiovascular disease, chronic kidney disease and diabetes

Hypothyroid participants were further divided into treated (receiving thyroid hormone replacement therapy) and untreated groups. However, the hyperthyroid treated group was too small in number to be analyzed as a separate subgroup. After multiple imputation for missing covariates (< 10% missing), we performed binary logistic regression analyses. After adjusting for all covariates listed in Table 7, participants receiving thyroid hormone replacement therapy had a significantly higher risk of a PHQ-9 score ≥ 5 than those with normal thyroid function (OR = 1.328; 95% CI: 1.115–1.582; *P* = 0.002). In contrast, the risk of a PHQ-9 score ≥ 5 did not significantly differ between untreated participants and those with normal thyroid function following adjustment for confounders (OR = 0.893; 95% CI: 0.589–1.356; *P* = 0.597). Furthermore, after adjustment for all covariates, the risk of having a PHQ-9 score ≥ 5 among hypothyroid patients receiving thyroid hormone replacement therapy was not significantly different from that among untreated hypothyroid patients with high TSH levels (OR = 1.486, 95% CI: 0.956–2.311; *P* = 0.078). Although the effect did not reach statistical significance (*P* = 0.078), it demonstrated a marginally significant trend at the α = 0.05 level. Meanwhile, as shown in Table 8, after excluding cases with missing thyroid function data, the treatment group had significantly lower TSH (2.93 ± 5.28 vs. 11.66 ± 15.20 mIU/L, *P* < 0.001) and TPOAb levels (71.26 ± 153.99 vs. 137.71 ± 296.87 IU/mL, *P* = 0.001), but significantly higher FT4 levels (0.97 ± 0.27 vs. 0.69 ± 0.16 ng/dL, *P* < 0.001), compared with the untreated group.

**Table 8:** TSH, FT4, TPOAb, and TgAb levels in the two hypothyroid patient groups

Overall	Hypothyroid without taking medication	Hypothyroid with taking medication	*p*-value
	133	465	
TSH (mIU/L)	11.66(15.20)	2.93(5.28)	< 0.001
FT4 (ng/dL)	0.69(0.16)	0.97(0.27)	< 0.001
TPOAb (IU/mL)	137.71(296.87)	71.26(153.99)	0.001
TgAb (IU/mL)	71.08(266.64)	55.68(240.37)	0.549

### Identification of DEGs

**Fig. 3:**
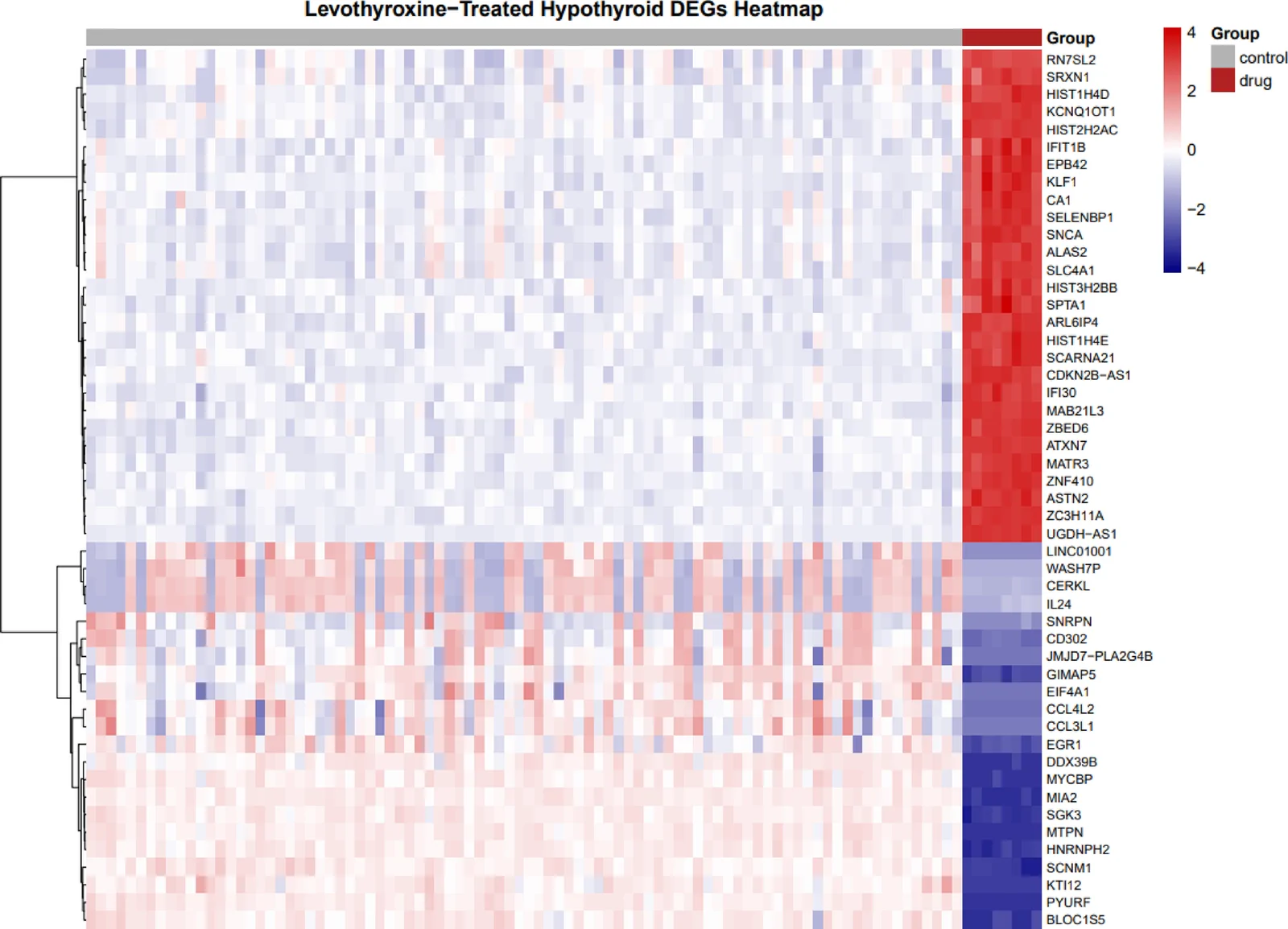
A heat map of LT4-treated Hypothyroidism DEGs analysis results based on the merged GSE103305 dataset

**Fig. 4:**
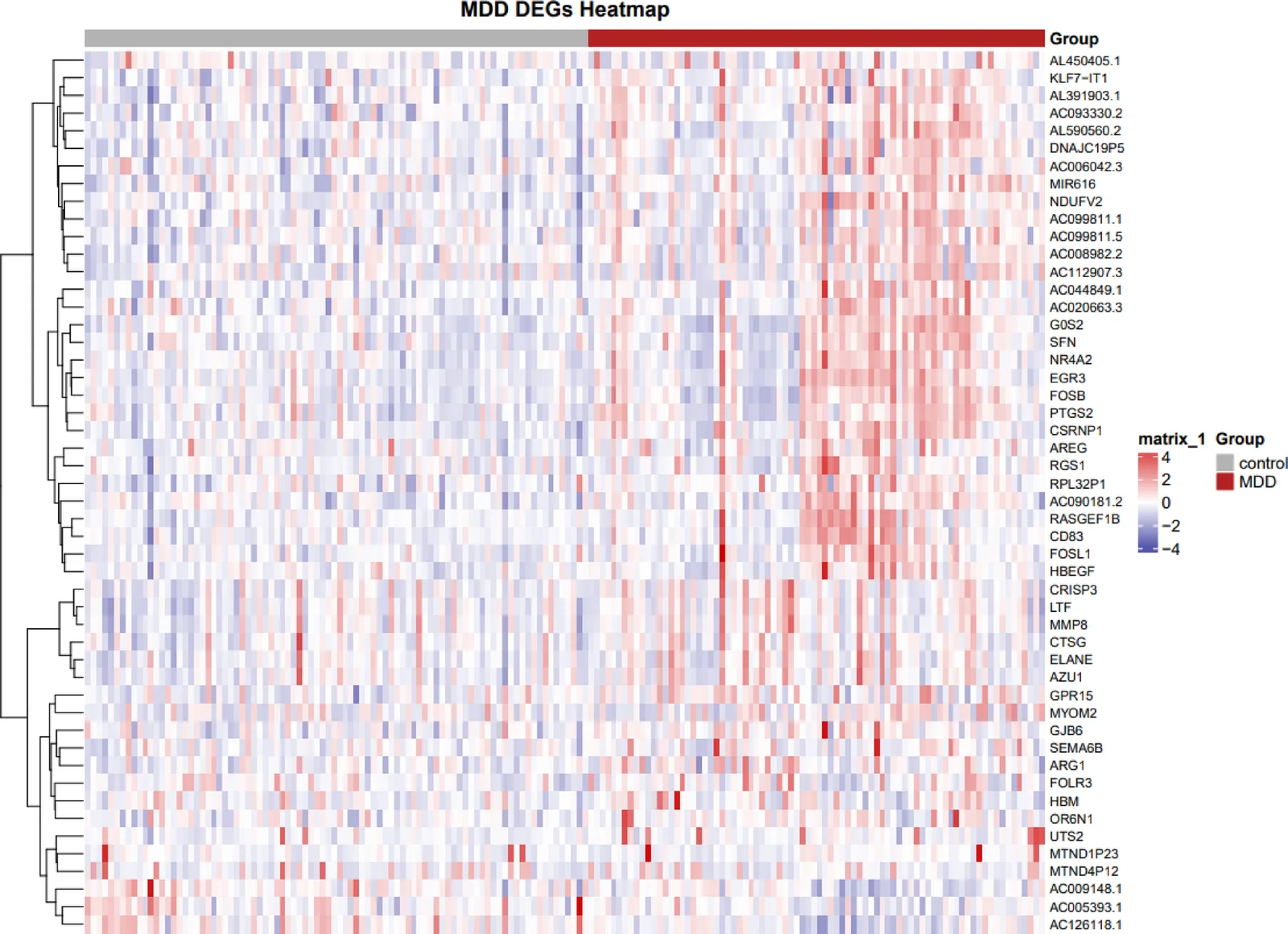
A heat map of MDD DEGs analysis results based on theGSE251778 dataset

**Fig. 5:**
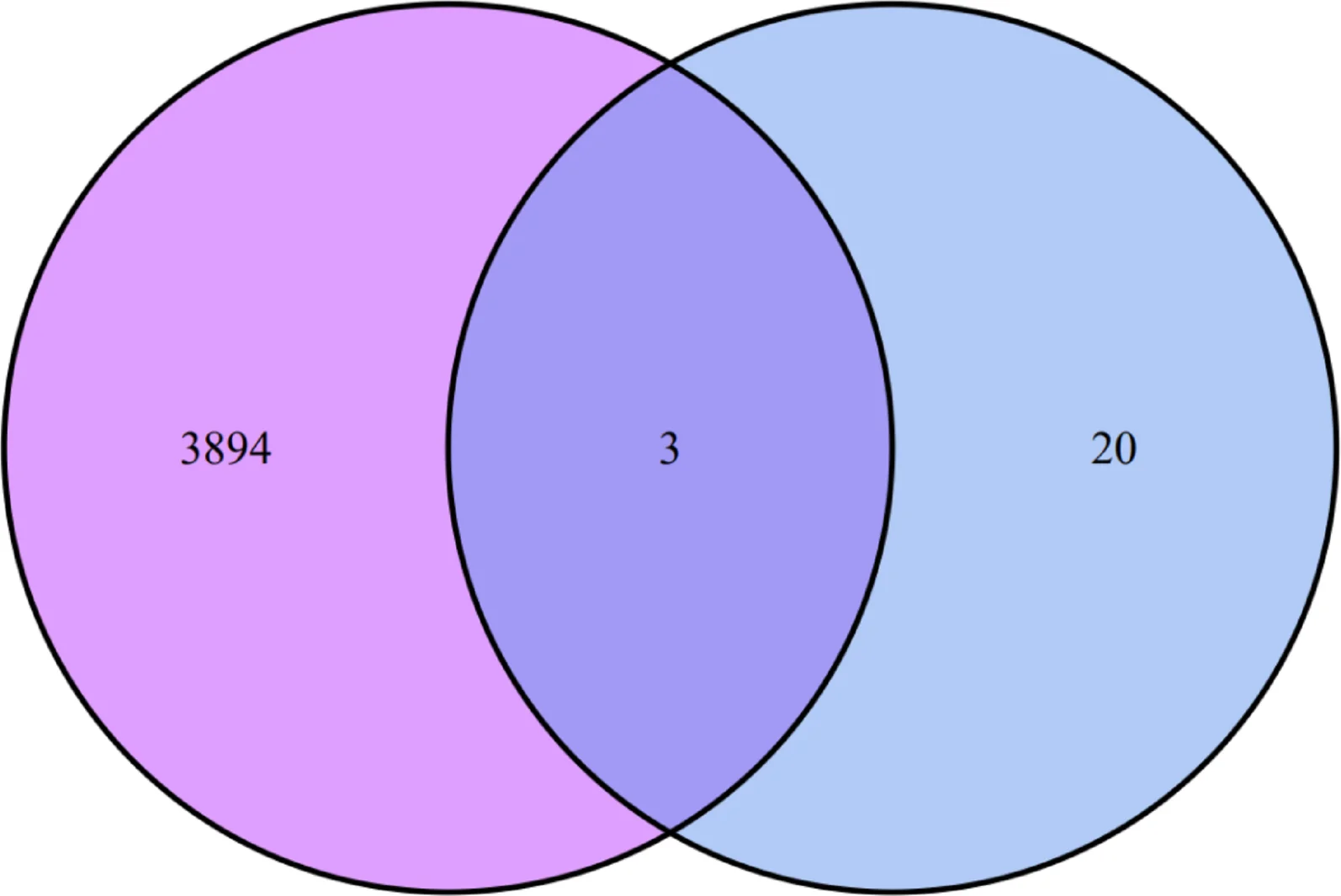
Common Downregulated Genes in Hypothyroidism(with LT4) and MDD

**Fig. 6:**
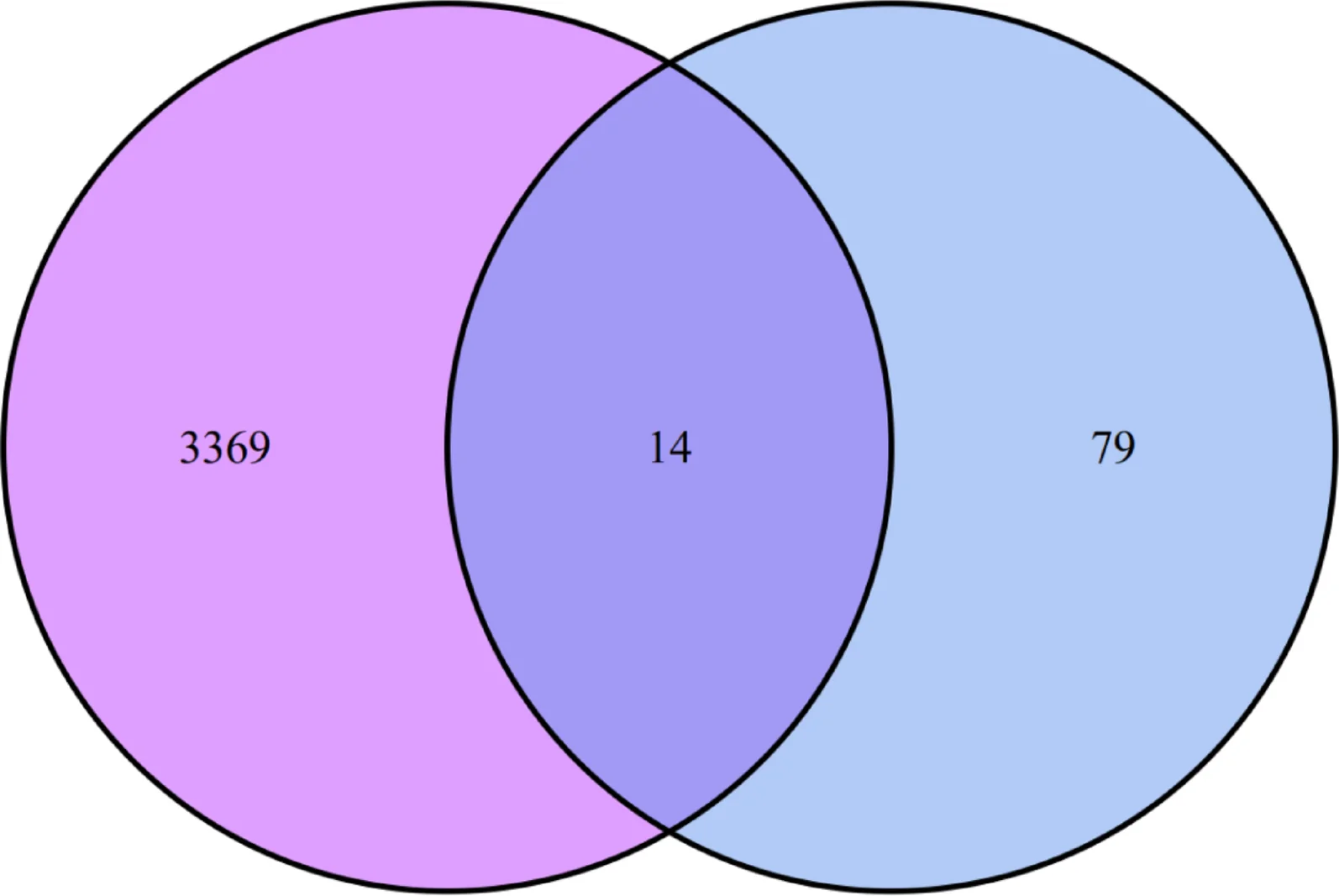
Common Upregulated Genes in Hypothyroidism(with LT4) and MDD

The hypothyroidism dataset from the GEO database contained 33,126 genes from 8 whole blood samples taken from 8 patients with hypothyroidism after supplementation with LT4. A total of 7,280 DEGs related to hypothyroidism after LT4 supplementation were identified in these datasets, of which 3,897 genes were downregulated and 3,383 genes were upregulated, as shown in the heat map (Fig. 3). We obtained 116 MDD-related DEGs in the GSE251778 dataset, of which 23 genes were downregulated and 93 genes were upregulated, as shown in the heat map (Fig. 4). The 17 common DEGs identified between the LT4-treated hypothyroidism group and the MDD group are represented by a Venn diagram, with 3 genes commonly downregulated (Fig. 5) and 14 genes commonly upregulated (Fig. 6).

### GO and KEGG enrichment analyses

**Fig. 7:**
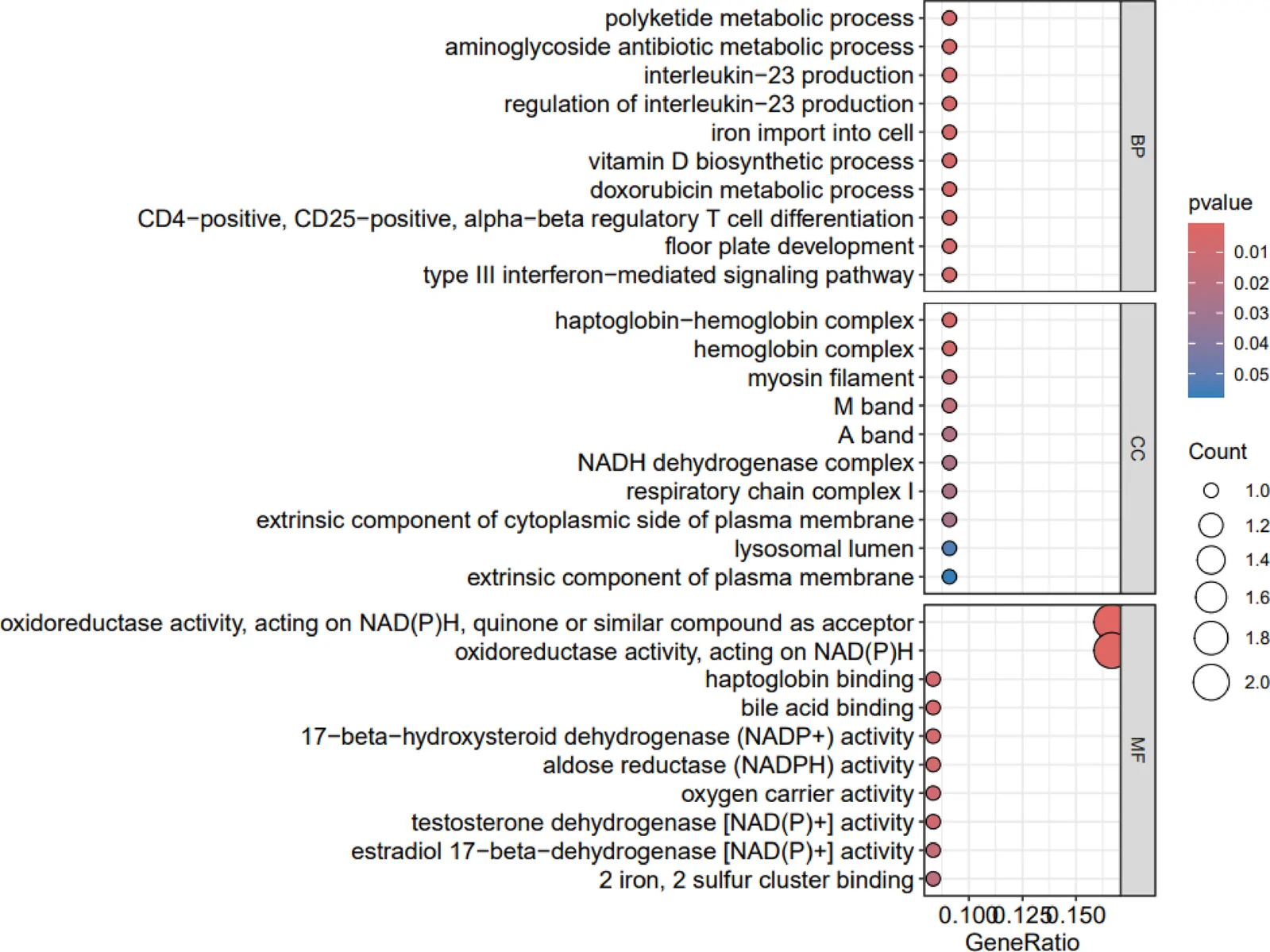
Gene ontology enrichment analysis of genes. GO, Gene Ontology

GO and KEGG pathway enrichment analyses were conducted to investigate the biological features and related pathways associated with the 17 shared DEGs. GO organizes biological information into three independent but interconnected ontologies: cellular component (CC), molecular function (MF), and biological process (BP). At the core of GO are terms, each of which describes a gene or protein’s function, cellular location, or biological process. These GO terms are connected by well-defined relationships, creating a hierarchical database. The GO enrichment analysis revealed significant enrichment in the MF category, specifically for the terms ‘oxidoreductase activity, acting on NAD(P)H, quinone or similar compound as acceptor’ and ‘oxidoreductase activity, acting on NAD(P)H’. No significant enrichment was observed in the CC or BP categories (Fig. 7). Despite the statistical significance of the p values for these two MF terms, their biological significance warrants further investigation because of the limited number of genes involved.

**Fig. 8:**
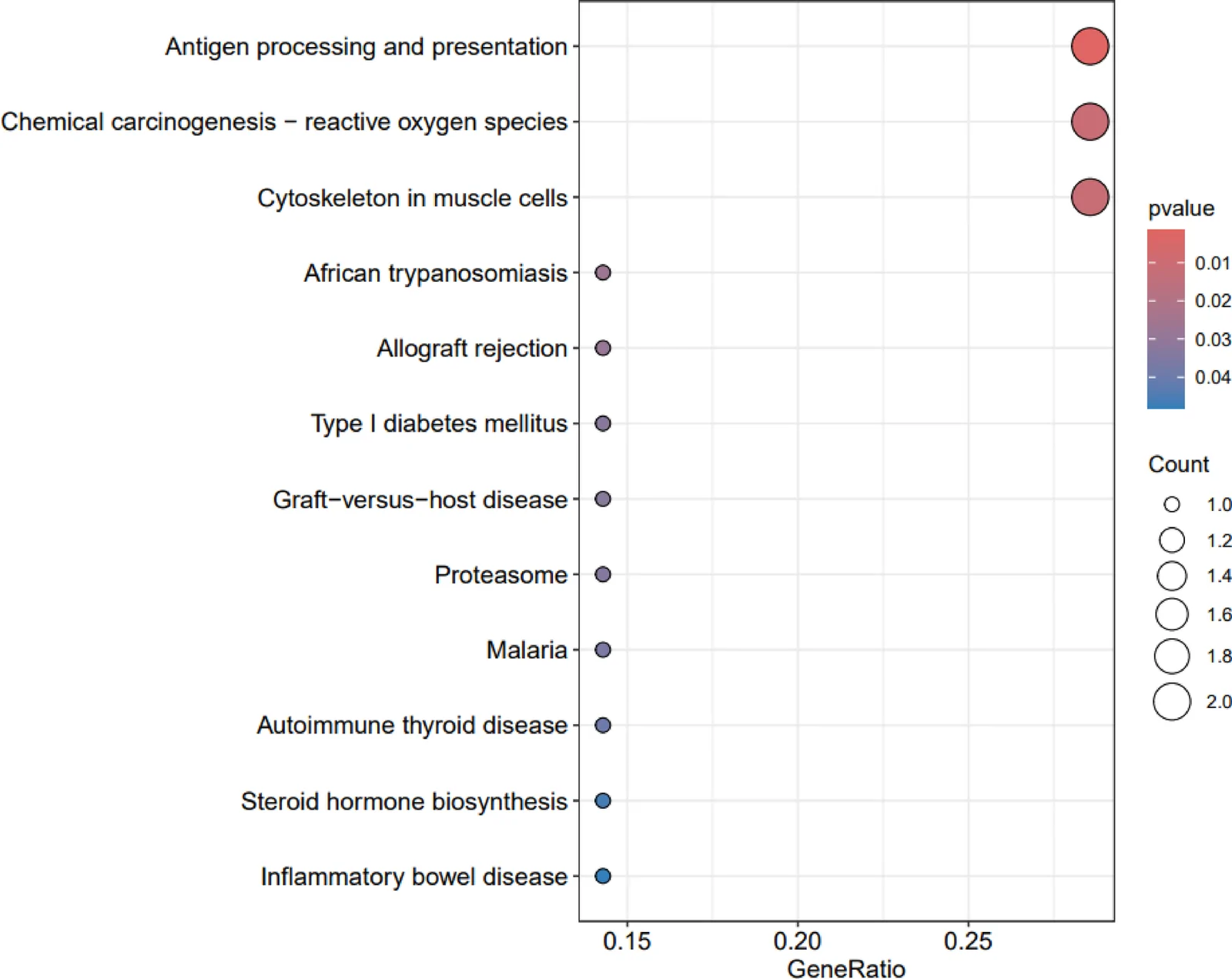
KEGG enrichment analysis of genes. KEGG, Kyoto Encyclopedia of Genes and Genomes

The KEGG database is designed for analysing genomic information and studying biological pathways and metabolic networks. Furthermore, a key challenge in the current research field is how to use genetic information to construct computational models for predicting complex cellular and organismal behaviours. The results of the KEGG enrichment analysis revealed the following: antigen processing and presentation, chemical carcinogenesis−reactive oxygen species and the cytoskeleton in muscle cells (Fig. 8).

### PPI network

**Fig. 9:**
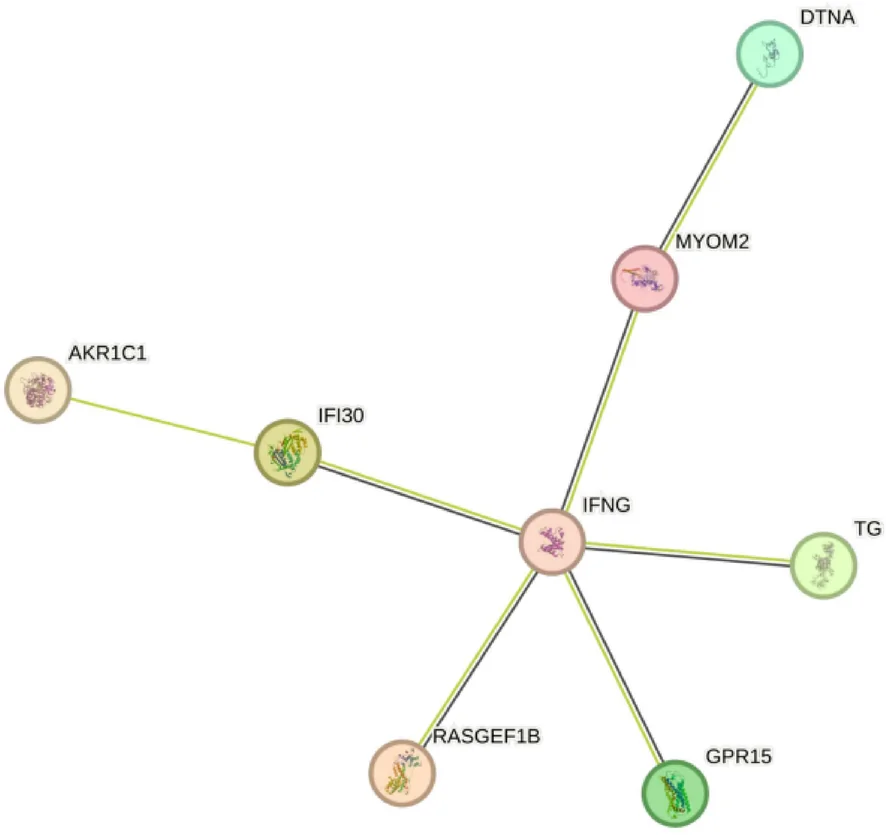
PPI network construction of key genes using STRING database. PPI, protein-protein interaction; STRING, Search Tool for the Retrieval of Interacting Genes/Proteins

The PPI network, as obtained from the STRING database, is shown in Fig. 9. With a combined score threshold of > 0.15, the network comprises 8 nodes and 13 edges. Each node represents a protein corresponding to a gene, with interacting nodes connected by edges. The more edges surrounding a node, the greater the likelihood of interaction. Based on the analysis, IFNG was identified as the gene with the highest degree.

## Discussion

This study was aimed at assessing the association between depressive symptoms and thyroid function in adults, with a focus on exploring the impact of thyroid hormone therapy on depressive symptoms prognosis and its underlying mechanisms. The thyroid gland is the largest endocrine organ in the human body and regulates growth, development, and metabolism through the synthesis and secretion of thyroid hormones [27]. Research has indicated that thyroid function plays a critical role in mood disorders, and even minor disruptions can lead to unstable emotional states [11, 28]. This effect may be mediated through the regulation of neurotransmitter levels such as serotonin (5-HT) and norepinephrine (NE) [13, 28]. Multiple animal studies have confirmed this phenomenon. In experimentally induced hypothyroid adult rat models, increased 5-HT turnover in the brainstem and decreased 5-HT concentration in the cortex were observed [1, 29]. Conversely, hyperthyroid models demonstrated elevated 5-HT levels but reduced NE concentrations [30]. Collectively, these findings indicate that thyroid dysfunction significantly affects monoamine neurotransmitter levels in the cerebral cortex.

However, the thyroid gland is regulated primarily by the hypothalamic-pituitary-thyroid (HPT) axis, which also plays a central role in the pathophysiology of depression [31–33]. Patients with depression exhibit HPT axis dysfunction, potentially interfering with thyroid function and resulting in a range of thyroid abnormalities. A systematic review supported this view, indicating that psychological stress (e.g., depression) can affect thyroid function via the HPT axis [34]. This study further revealed that the course of depression (including the duration and frequency of episodes) influences thyroid hormone levels in hair.

In our analysis of the NHANES data, we grouped participants by thyroid function status and demonstrated a significant positive correlation between hypothyroidism and total PHQ-9 scores in adults. Compared with individuals without depressive symptoms, individuals with mild or moderate depression were more likely to have hypothyroidism. However, when individuals with hyperthyroidism were compared to those with normal thyroid function, the only significant finding was that individuals with mild depression were more likely to have hyperthyroidism than those without depressive symptoms. These findings are consistent with those of previous research highlighting the potential association between thyroid dysfunction and mood disorders [7, 13, 33]. However, it should be emphasized that the number of participants diagnosed with hyperthyroidism in this study was relatively small, with particularly low numbers of patients with mild depression (*n* = 28), moderate depression (*n* = 8), and moderately severe/severe depression (*n* = 5) in the hyperthyroid group. These sample size limitations have affected the statistical power and accuracy of the results. Furthermore, hypothyroidism is correlated with specific symptoms of depression. Patients presenting with decreased interest, appetite changes (e.g., poor appetite or overeating), or feelings of worthlessness/failure are more likely to have hypothyroidism than those without these depressive symptoms. This association suggests that depressive symptoms and hypothyroidism may share common pathophysiological mechanisms. Accordingly, clinicians should prioritize thyroid function screening in patients with such symptoms.

The stratification of participants by total PHQ-9 scores revealed an increased risk of having a PHQ-9 score ≥ 5 in those with hypothyroidism. After adjusting for confounding factors including demographic information, family income-to-poverty ratio, exercise, and comorbidities, individuals receiving thyroid hormone replacement therapy had a greater risk of having a PHQ-9 score ≥ 5 compared to those with normal thyroid function. In contrast, no significant difference was observed in the risk of having a PHQ-9 score ≥ 5 between hypothyroid individuals not receiving medication and those with normal thyroid function. Compared with the untreated group, the treatment group had lower mean TSH and TPOAb levels and a higher mean FT4 level, suggesting less severe autoimmune damage and better thyroid function. However, it unexpectedly showed a trend toward a higher risk of PHQ-9 score ≥ 5. This phenomenon was not isolated. For instance, a large community-based, controlled survey in 2002 demonstrated that compared with age- and sex-matched controls, patients on thyroxine replacement, even those with normal TSH levels, had significantly impaired psychological well-being [35]. A 2018 retrospective cross-sectional study also revealed persistent impairment in cognitive function and general well-being in patients who underwent long-term LT4 replacement [36]. The above evidence was consistent with the findings of our study. This suggests that although thyroid hormone replacement therapy can effectively normalize biochemical indicators of thyroid function, it does not appear to confer corresponding mental health benefits, and may even be associated with an increased risk of depressive symptoms. Nevertheless, this association should be interpreted with caution. On the one hand, previous studies have shown an association between autoimmune thyroid disease (AITD) and mood disorders (including depression), with anti-thyroid antibody levels being negatively correlated with quality of life [2, 3, 37, 38]. Although the treatment group in this study had relatively lower antibody levels, they still exceeded the normal range for TPOAb (0–9.0 IU/mL) and TgAb (0–4.0 IU/mL), respectively [39–44]. Thus, a direct contribution of autoimmune factors to depressive symptoms cannot be entirely ruled out. On the other hand, that the treatment group received hormone replacement therapy may indicate more severe baseline disease. But the absence of baseline thyroid function data at diagnosis in this study precludes verification of this inference. In summary, limited by the cross-sectional design and sample size, this study could not definitively disentangle the independent contributions of thyroid hormone replacement therapy, autoimmune damage, and initial disease severity to depressive symptoms. However, this trend suggests that there may be an association worthy of further exploration between thyroid hormone replacement therapy and depressive symptoms, and future studies using methods such as large-sample longitudinal follow-up data are needed for further validation.

In the second part of this study, we identified common DEGs between hypothyroid patients receiving LT4 therapy and patients with MDD, and performed GO/KEGG enrichment analysis and PPI network analysis, providing preliminary clues for the molecular association between the two groups. GO enrichment analysis revealed significant enrichment of oxidoreductase-related terms, such as “oxidoreductase activity, acting on NAD(P)H”, among the 17 common DEGs, with 14 genes being upregulated. KEGG pathway analysis also identified enrichment in reactive oxygen species (ROS)-related pathways, such as “chemical carcinogenesis-reactive oxygen species”. These results suggest that redox homeostasis may be dysregulated in both diseases, and that oxidative stress may be involved in their shared pathophysiological processes. Under conditions of oxidative stress, excessive ROS can interfere with signal transduction, damage biomolecules, and trigger inflammatory and immune responses [45]. Notably, endogenous ROS mainly originate from the mitochondrial respiratory chain and enzymes such as NADPH oxidase [46]. Brain neurons, astrocytes, and microglia are rich in mitochondria and NADPH oxidases; however, the antioxidant capacity of the brain is relatively restricted owing to its high lipid content and intense oxidative metabolism [45, 47, 48]. Therefore, upregulation of oxidoreductase-related genes may confer a higher susceptibility to oxidative damage in the brain, which might partially explain the shared basis of brain dysfunction in both diseases.

The above findings are in line with previous reports that oxidative stress is involved in the pathogenesis of depression, involving mechanisms such as neuroinflammation and mitochondrial dysfunction, suggesting that antioxidant pathways may represent one of the potential strategies for the prevention and treatment of depression [49]. In addition, previous meta-analyses have indicated that patients with hypothyroidism exhibit mild systemic inflammation and elevated levels of systemic oxidative stress [50]. Although LT4 replacement therapy can alleviate the immune-inflammatory response in hypothyroid patients, its effect on oxidative stress levels remains inconclusive [51]. A U.S. survey on adverse reactions to LT4 treatment revealed that more than 20% of treated patients may experience side effects of iatrogenic hyperthyroidism, such as agitation/anxiety, which had a linear correlation with plasma hydroperoxides (a measure of oxidative stress) [52, 53]. Specifically, the peak levels of thyroxine (T4) and triiodothyronine (T3) occurring 1 to 2 h after LT4 administration can trigger an increase in plasma hydroperoxides, whereas the intake of physiological regulators with antioxidant activity significantly reduces this trend [54, 55]. Conversely, some studies have indicated that LT4 replacement therapy can significantly reduce malondialdehyde (a biomarker of oxidative stress) levels and increase catalase (an antioxidant enzyme) activity, thereby improving the oxidative status in patients with hypothyroidism [56]. In summary, although LT4 is widely used to treat hypothyroidism, its specific effect on oxidative stress levels remains inconclusive. Based on the analytical results of this study, we speculate that LT4 replacement therapy may instead increase systemic oxidative stress levels; however, this speculation still requires verification through animal experiments and prospective clinical studies.

Furthermore, KEGG analysis also revealed the enrichment of the “cytoskeleton in muscle cells” pathway, suggesting that LT4 may indirectly influence the stress response and emotional states by affecting genes associated with the muscle cell cytoskeleton. This is because skeletal muscle, which is the largest tissue in the human body, may be involved in metabolism, inflammation, and stress responses.

According to the results of the PPI network analysis, the expression of IFNG, the gene with the highest degree of interaction, was downregulated in both groups. The IFNG gene primarily encodes interferon-gamma (IFN-γ), an important multifunctional cytokine that plays a central role in the immune system and inflammatory responses. Lee et al. investigated medication-naive adolescents with first-episode MDD and reported lower levels of IFN-γ in untreated adolescents with MDD [57]. Bayes et al. performed a subtype classification of patients with MDD and reported reduced levels of IFN-γ in patients with two subtypes of MDD, which is consistent with our findings [58]. However, the literature concerning the effects of thyroid hormone replacement therapy on serum cytokines is relatively scarce. Guclu et al. reported no statistically significant reduction in serum IFN-γ levels in hypothyroid patients with Hashimoto’s thyroiditis who were treated with LT4 for 12 weeks [59]. Wei et al. examined IFN-γ expression levels in rats from an experimentally thyroidectomized group treated with T4 and reported significantly elevated levels of both IFN-γ mRNA and protein [60]. Future research could further explore the potential impact of thyroid hormone replacement therapy on serum cytokines, including IFN-γ.

Based on NHANES epidemiological survey data and GEO gene expression data, this study establishes a multilevel and systematic research framework to explore the complex relationship between depressive symptoms and thyroid dysfunction in depth as well as the underlying molecular mechanisms. The strengths of this study lie in the fact that all the sample data for the first part were sourced from the U.S. NHANES public database, which has standardized and rigorous data collection processes, ensuring good traceability, reliability, and national representativeness. Moreover, a bidirectional logistic regression analysis was performed to investigate the potential reciprocal relationships between depressive symptoms and thyroid dysfunction comprehensively. Particular attention was given to comparing depression risk differences among hypothyroid patients receiving LT4 treatment, healthy controls, and untreated hypothyroid patients, revealing significant associations within specific clinical contexts. The data for the second part were entirely sourced from the GEO database, in which only high-impact articles from Q1 and Q2 journals (according to JCR classification) were selected, thereby ensuring the quality of the data and the reliability of the study design as much as possible. By successfully integrating clinically observed findings from the NHANES with gene expression data from the GEO database, this study further elucidated potential common biological pathways and molecular networks that may simultaneously drive the pathophysiological processes of both thyroid dysfunction and depressive symptoms. This systematic exploration, spanning from macro clinical phenotypes to micromolecular mechanisms, offers new insights into the underlying biology of the comorbidity for these two diseases and is expected to provide molecular targets for relevant clinical diagnosis and treatment strategies.

While this study demonstrates certain advantages in terms of data integration and multifaceted analysis, several limitations should be acknowledged. First, the cross-sectional nature of NHANES data primarily reveals associations between variables rather than establishing causal relationships, and the findings were subject to unmeasured confounding factors, such as pre-treatment illness severity and antidepressant use. The depressive symptoms assessment relied solely on questionnaire-based evaluations without supplementary medical records or auxiliary clinical examinations, which might introduce outcome bias. Additionally, depressive symptoms were defined using a PHQ-9 cut-off of ≥ 5 in this study; therefore, our findings are limited to depressive symptoms and should not be extrapolated to clinical depression. Second, the GEO datasets may have limitations in terms of the number of DEGs and the sample size, potentially constraining the analytical power; thus, the results require validation in larger, independent cohorts. Lastly, the pathways and core genes identified through GO, KEGG, and PPI analyses were limited in number, and some findings were based on database predictions. Although these analyses suggest multiple potential biological clues, the specific functional roles and causal mechanisms of these candidates in disease pathogenesis and progression need further confirmation through in vivo and in vitro experiments.

## Conclusions

Based on an analysis of the NHANES database, this study confirmed a bidirectional association between thyroid disease (particularly hypothyroidism) and depressive symptoms. Notably, for the first time in a general population, we identified a potential association between LT4 replacement therapy and an increased risk of depressive symptoms in patients with hypothyroidism. To explore the underlying mechanisms, we further analyzed GEO datasets, and the results suggested that disruptions in redox homeostasis and IFN-γ signaling pathways might represent one of the molecular mechanisms involved. These findings provide new perspectives for understanding the pathophysiological basis of comorbid thyroid dysfunction and depressive symptoms, and may offer some insights for future exploration of individualized treatment strategies.

## Data Availability

Publicly available datasets were analyzed in this study. NHANES data can be found: https://www.cdc.gov/nchs/nhanes/. GEO data can be found: https://www.ncbi.nlm.nih.gov/geo/.
